# Baseline Characteristics of Adult Patients Treated and Never Treated with Teduglutide in a Multinational Short Bowel Syndrome and Intestinal Failure Registry

**DOI:** 10.3390/nu16152513

**Published:** 2024-08-01

**Authors:** Gabriel E. Gondolesi, Ulrich-Frank Pape, Joel B. Mason, Johane P. Allard, Loris Pironi, María Núria Virgili Casas, Lauren K. Schwartz, Francisca Joly, André Gabriel, Sasan Sabrdaran, Pinggao Zhang, Martina Kohl-Sobania, Yi-Wen Huang, Palle B. Jeppesen

**Affiliations:** 1 Intestinal Failure, Rehabilitation and Transplant Unit, Hospital Universitario Fundación Favaloro, Buenos Aires C1044AAA, Argentina; 2 Asklepios Klinik St. Georg, Asklepios Medical School, 20099 Hamburg, Germany; 3 Department of Hepatology and Gastroenterology, Charité University Medicine Berlin, 13353 Berlin, Germany; 4 Tufts Medical Center, Tufts University, Boston, MA 02111, USA; 5 Toronto General Hospital, University of Toronto, Toronto, ON M5G 2C4, Canada; 6 Department of Medical and Surgical Sciences, University of Bologna, 40138 Bologna, Italy; loris.pironi@unibo.it; 7 Centre for Chronic Intestinal Failure, IRCCS Azienda Ospedaliero-Universitaria di Bologna, 40138 Bologna, Italy; 8 Endocrinology and Nutrition Department, Hospital Universitari de Bellvitge, 08907 Barcelona, Spain; 9 NYU Langone Medical Center, New York, NY 10016, USA; 10 Hôpital Beaujon, University of Paris Cité, 92110 Clichy, France; 11 Takeda Development Center Americas, Inc., Cambridge, MA 02142, USA; 12 Outpatient Clinic, Pediatric Emergency Department, University Hospital Schleswig-Holstein, 23538 Lübeck, Germany; 13 Rigshospitalet, Copenhagen, Denmark; palle.bekker.jeppesen@regionh.dk

**Keywords:** short bowel syndrome, intestinal failure, teduglutide, GLP-2 analogs, parenteral nutrition, Crohn’s disease, real-world, colorectal polyps, Gattex, Revestive

## Abstract

The Short Bowel Syndrome (SBS) Registry (NCT01990040) is a multinational real-world study evaluating the long-term safety of teduglutide in patients with SBS and intestinal failure (SBS-IF) in routine clinical practice. This paper describes the study methodology and baseline characteristics of adult patients who have (ever-treated) or have never (never-treated) received teduglutide. A total of 1411 adult patients (679 ever-treated; 732 never-treated) were enrolled at 124 sites across 17 countries. The mean (standard deviation [SD]) age at enrollment was 55.4 (15.46) years, and 60.2% of patients were women. Crohn’s disease was the most common cause of major intestinal resection in both ever-treated (34.1%) and never-treated patients (20.4%). A similar proportion of ever-treated and never-treated patients had a prior history of colorectal polyps (2.7% vs. 3.6%), whereas proportionally fewer ever-treated patients reported a history of colorectal cancer (1.8% vs. 6.2%) or any malignancy (17.7% vs. 30.0%) than never-treated patients. Never-treated patients received a numerically greater mean (SD) volume of parenteral nutrition and/or intravenous fluids than ever-treated patients (12.4 [8.02] vs. 10.1 [6.64] L/week). Ever-treated patients received a mean teduglutide dosage of 0.05 mg/kg/day. This is the first report of patient baseline characteristics from the SBS Registry, and the largest cohort of patients with SBS-IF to date. Overall, ever-treated and never-treated patients had similar baseline characteristics. Differences between treatment groups may reflect variations in patient selection and degree of monitoring.

## 1. Introduction

Short bowel syndrome (SBS) is a chronic malabsorptive disorder and the most frequent cause of intestinal failure (IF) in both adults and children [1,2,3]. Patients with SBS and intestinal failure (SBS-IF) are reliant on parenteral nutrition and/or intravenous fluids (PN/IV) to provide their nutrition and hydration requirements to maintain health and growth [3]. Although PN/IV can be lifesaving, long-term administration has been associated with potentially life-threatening complications, such as liver disease, loss of central venous access, venous catheter-related bloodstream infections, and sepsis [2,3,4,5,6,7].

Glucagon-like peptide (GLP-2), an intestinotrophic pro-adaptive factor, enhances nutrient absorption and stimulates proliferation and adaptation of the intestinal epithelium [8,9,10]. Teduglutide is a semisynthetic analog of the GLP-2 molecule that is approved for the treatment of adult and pediatric patients with SBS-IF [11] in several territories, including Europe [12,13,14], North America (including Canada [15] and the USA [11]), South America (including Argentina [16], Brazil [17], and Chile [18]), Japan [19], and Australia [20]. A single amino acid substitution present in teduglutide makes the molecule resistant to degradation and so extends its half-life and increases its potency when compared with native GLP-2 [21,22]. In short- and medium-duration trials, teduglutide has been shown to enhance intestinal adaptation in patients with SBS-IF, reduce dependence on PN/IV [23,24,25], and has had its safety and efficacy established in phase 3 clinical trials [24,26,27,28,29,30,31,32,33,34]. In addition, the effectiveness of teduglutide in reducing dependence on PN/IV and improving stool characteristics were reported in real-world studies [35,36,37,38,39]. Patients receiving teduglutide are advised to have periodic colonoscopies [11,40,41] owing to the intestinotrophic properties of this treatment and the hypothesized risk of inducing or enhancing growth of neoplastic tissue within the gastrointestinal (GI) tract, such as precancerous polyps (colon adenomas, sessile serrated polyps) or colon cancer [42]. To date, however, the development of these cancers has only been reported for individual cases of patients receiving teduglutide [43,44].

It is difficult to generalize the clinical presentation and treatment course of patients with SBS-IF based on clinical data, owing to the heterogeneous characteristics of SBS-IF [45]. Real-world studies provide an opportunity to collect long-term data in a large, diverse population and to develop an understanding of the safety and effectiveness of therapies in real-life clinical practice [46,47].

The SBS Registry (NCT01990040) is a global, prospective, observational, multicenter effort to evaluate the risk of colorectal cancer in adult and pediatric patients with SBS-IF treated with teduglutide [48]. The study is a regulatory commitment for both the European Medicines Agency (EMA) and the US Food and Drug Administration (FDA), with a 10-year follow-up target for each enrolled patient.

This paper describes the development of the SBS Registry and provides the baseline characteristics of adult patients who have been enrolled. Patient outcomes will be reported in subsequent publications.

## 2. Methods

### 2.1. Study Design Overview

The SBS Registry recruited patients with SBS-IF who have received or have never received teduglutide and collects data on the clinical outcomes and potential risks of teduglutide in a real-world setting. Patient enrollment is now complete, and patient baseline characteristics have been analyzed.

All patients were enrolled according to Protocol Version 6.0 (dated 5 August 2019), in which patients must have been receiving PN/IV for at least 6 months at the time of enrollment to be considered as having IF. Data will be collected over a minimum 10-year follow-up period. Patients may participate as long as the registry is active and may withdraw at any time. The registry includes both adult and pediatric patients, although this paper only describes the adult population; data on pediatric patients will be provided in future publications.

### 2.2. Study Objectives

The primary objective of this registry is to evaluate the long-term safety profile of teduglutide when treating adult and pediatric patients with SBS-IF in a routine clinical setting. The primary safety outcome is the occurrence of colorectal cancer in patients with SBS-IF who have any remnant colon and have received teduglutide. The secondary objectives are to evaluate the long-term clinical outcomes in patients with SBS-IF who are treated with teduglutide in a routine clinical setting versus patients who have never received teduglutide. Data on the analyses of the primary and secondary outcomes will be provided in future publications. Here, we report the baseline characteristics and demographics of the patients enrolled in the registry.

### 2.3. Patient Recruitment

Healthcare providers treating patients with SBS-IF could participate at study sites on a voluntary basis if their patients were enrolled through previous clinical trial treatment clinics or if they had access to claims and/or medical records to identify patients with SBS-IF. Eligible patients were male or female of any age, had a diagnosis of SBS-IF, and had been receiving PN/IV for at least 6 months at the time of enrollment (Supplementary Table S1). Patients who were 18 years of age or older were categorized as adults. This registry planned to recruit as many patients with SBS-IF as possible across multiple countries, with the goal of enrolling approximately 1310 adult patients.

For data analysis, patients were divided into two groups based on their exposure to teduglutide: ‘ever-treated’ (patients who had received at least one dose of teduglutide before or during the registry) and ‘never-treated’ (patients who had never received teduglutide and did not receive teduglutide during this study period). Both groups continued to receive the usual standard of care. If patients qualify to start using teduglutide, they can move from the ‘never-treated’ to the ‘ever-treated’ group. However, patients in the ‘ever-treated’ group who stop receiving teduglutide will remain in the ever-treated group.

### 2.4. Data Collection

Data from patients’ medical records and notes were collected at baseline and will be recorded at least every 6 months during the follow-up period. The patient demographics recorded included age, gender, race, ethnicity, height, weight, and body mass index (BMI). History of SBS-IF was recorded at enrollment, including age at onset/diagnosis; cause of major intestinal resection; colon anatomy (intact, remnant colon, no colon); length of remaining colon and small intestine when available; and the presence of stoma. Past medical history was also recorded, including the presence of colorectal cancers; malignancy of any other type; colorectal polyps; benign neoplasia of the GI tract and other GI conditions; and duration of PN/IV support received before enrollment. Exposure to teduglutide was recorded for ever-treated patients, including how long they had received teduglutide before enrollment and the dosage received.

During the follow-up period, this study will continue to evaluate safety (including occurrence of other malignancies, benign neoplasia of the GI tract/hepatobiliary system/pancreas, presence of colorectal polyps, and occurrence of adverse events potentially related to treatment with teduglutide) and effectiveness (including changes between baseline and follow-up visits in PN/IV volume, number of days per week receiving PN/IV, and proportion of patients weaned off PN/IV) outcome variables. Adverse event reporting is summarized in Supplementary Materials S1. The results of these assessments will be reported in future papers.

### 2.5. Data Analysis

Patients will be analyzed under discrete outcome sets. The per protocol set (PPS; all patients who met the protocol inclusion and exclusion criteria) is the group described in this paper. The primary outcome will be assessed in the primary outcome analysis set (POS; a subset of the PPS who are adult patients with any remnant colon and without colorectal cancer at baseline). Finally, effectiveness outcomes will be assessed in the effectiveness analysis set (EAS; a subset of the PPS that includes all never-treated patients and data from ever-treated patients before discontinuation of teduglutide, patient death/withdrawal/other loss to follow-up, site closure [without transfer to another site in the study], last known contact or data cutoff date for interim analyses).

### 2.6. Statistical Analysis of Demographic and Baseline Characteristics

Descriptive statistics were used to summarize the data collected for demographic and baseline characteristics, SBS-IF history, baseline medical history, and teduglutide exposure. No formal statistical testing was performed on the baseline data because the objective of this analysis is to describe the development of the SBS Registry and provide the baseline characteristics of the adult patients who have enrolled; consequently, only numerical differences are recorded in this paper. The effects of the treatments occur after baseline, and therefore, any disparities observed in the baseline data can be predominantly attributed to pre-existing selection biases rather than the influence of the treatments. Overall, outcomes will be summarized as the number of patients, percentage, and mean and standard deviation (SD) of each categorical and continuous outcome variable. Baseline data were collected at the time of study enrollment. For ever-treated patients who received teduglutide prior to enrollment, the baseline PN/IV characteristics and cases of colorectal cancer were collected at the time point immediately before the first dose of teduglutide. The number of patients who were likely to be enrolled was estimated using a simulation approach. The assumptions made for the simulation study, and calculations for the rates of annual attrition and percentages of new teduglutide patients who would enroll in the study, are provided in Supplementary Materials S2 and S3. Based on these calculations, it was estimated that enrolling a population in which 30% of the patients with any remnant colon who had recently initiated treatment with teduglutide (*n* = 393), combined with an annual attrition rate of 15%, would yield a total of 2072 person-years (PYs) of exposure and a background risk for colorectal cancer of 120.1 cases per 100,000 PYs.

### 2.7. Planned Statistical Analysis of Outcomes

Analyses of the primary and secondary outcomes will be provided in future publications. The primary objective will be assessed using the incidence of confirmed colorectal cancer. Standardized morbidity ratios (SMRs) will be applied to examine whether there is a statistically significant difference in the incidence rate of colorectal cancer between ever-treated patients with any remnant colon in this study and the incidence rates from US and global cancer data sources (Surveillance Epidemiology and End Results [SEER] and the World Health Organization [WHO] Global Cancer Observatory [GLOBOCAN]). The null hypothesis is SMR ≥ 3.1 in the general population based on SEER and GLOBOCAN data, and the alternative hypothesis is SMR < 3.1. This calculation takes into account variables including the patients’ age, gender, length of follow-up, and presence of Crohn’s disease. This analysis may also be performed for the never-treated cohort, and the never-treated cohort will be employed as an internal comparison group, should sufficient data be available at the point of final analysis.

Other safety objectives will be analyzed through comparisons of incidence rate ratios and corresponding 95% confidence intervals between the ever-treated and never-treated patients using Poisson regression models, if necessary. Descriptive statistics for the effectiveness outcomes will be summarized using sample size, mean, SD, median, 25th percentile (Q1), 75th percentile (Q3), minimum and maximum; these will be presented during follow-up as both annual and cumulative analyses. In addition, effectiveness objectives will be analyzed using a mixed-effects repeated measures model (MMRM) with an unstructured covariance structure to measure changes in PN/IV volume between ever-treated and never-treated patients over time. The results of these effectiveness and safety analyses will be reported in subsequent papers.

### 2.8. Ethics

Each patient provided written informed consent before enrolling in the study. Regulatory approval of Protocol Version 6.0 was obtained from the EMA and FDA in January 2020 for all sites. All study protocols were approved by each individual institutional ethical committee and conducted in compliance with the International Conference on Harmonization Good Clinical Practice guidelines and the World Medical Association Declaration of Helsinki and its amendments. A de-identified data set will be available upon request for research purposes following final analysis of the data.

## 3. Results

### 3.1. Patient Demographics

Of the 851 study sites invited to participate, 153 study sites agreed and 144 (94%) study sites enrolled patients. Adult patients were enrolled from 124 sites. Between 23 June 2014 and 30 June 2022, 1411 adult patients (679 ever-treated; 732 never-treated) were enrolled across 17 countries (Table 1), of whom 1406 (677 ever-treated; 729 never-treated) met the inclusion criteria to participate in the registry. The mean (SD) age at enrollment was 55.4 (15.46) years; 60.2% of patients were women; and 72.0% were White. The mean (SD) height, weight, and BMI were 166.9 (10.11) cm, 63.8 (15.23) kg, and 22.8 (4.71) kg/m^2^, respectively. Half of the recruited patients were either retired or unable to work. Patient demographics and baseline characteristics are presented in Table 2.

### 3.2. SBS-IF History at Enrollment

Data on patient history of SBS-IF at enrollment (Table 3) are provided as mean (SD) values unless otherwise specified. Age at SBS-IF onset was 47.2 (18.39) years, and time between onset/diagnosis of SBS-IF and enrollment in the study was 8.2 (9.53) years. The most common cause of major intestinal resection was Crohn’s disease (ever-treated, 34.1%; never-treated, 20.4%), followed by intestinal ischemia (ever-treated, 15.4%; never-treated, 15.8%). Approximately half (55.4%) of enrolled patients had a stoma; ileostomy was the most common in both ever-treated and never-treated cohorts (61.2% and 46.3%, respectively), followed by jejunostomy (27.4% and 39.3%, respectively). The residual small intestine length was shorter in ever-treated (85.3 [55.54] cm) patients than never-treated (101.0 [73.17] cm) patients. The proportions of remnant colon in ever-treated (49.2 [24.01]%) and never-treated (56.7 [21.67]%) patients were similar.

### 3.3. Patient Medical History at Enrollment

Ever-treated and never-treated patients had similar frequencies of previous colorectal polyps (2.7% vs. 3.0%, respectively) and other benign neoplasia (of the GI tract [2.2% vs. 3.0%] of the hepatobiliary system [1.2% vs. 0.4%], and/or of the pancreas [0.3% vs. 0.8%]), as well as similar frequencies of intestinal obstruction (29.4% vs. 33.2%), IF-associated liver disease (11.5% vs. 10.8%), and central line infections (19.9% vs. 22.5%; Table 4). Ever-treated patients had a smaller proportion of colorectal polyps classified as adenomas, and a greater proportion classified as non-adenomas, than never-treated patients. A smaller proportion of ever-treated patients than never-treated patients reported a history of any malignancy (17.7% vs. 30.0%) and colorectal cancer (1.8% vs. 6.2%). Past history of colorectal cancer was reported in 12 (1.8%) ever-treated patients and 45 (6.2%) never-treated patients. The mean (SD) periods between end of colorectal cancer and initiation of teduglutide, or study inclusion, were 94.2 (30.5) and 62.2 (58.8) months for ever-treated and never-treated patients, respectively. Malignant neoplasms reported in ever-treated and never-treated patients included colon, breast, and cervical cancer. The mean (SD) periods between the end of malignancy and initiation of teduglutide, or study inclusion, were 56.8 (62.3) and 82.4 (93.6) months for ever-treated and never-treated patients, respectively.

### 3.4. Treatment Before Enrollment

Ever-treated patients had been receiving PN/IV for longer than never-treated patients (mean [SD] duration: 6.3 [6.86] years vs. 5.1 [6.15] years; PPS). Patients in the ever-treated cohort received a lower mean (SD) volume of PN/IV than never-treated patients (10.1 [6.64] vs. 12.4 [8.02] L/week) across approximately the same mean (SD) number of days/weeks (5.3 [1.76] vs. 5.7 [1.78] days/weeks; Table 5).

### 3.5. Teduglutide Exposure Status

Most of the ever-treated patients (65.3%) were already receiving teduglutide at enrollment and/or had started receiving teduglutide outside of a clinical trial (69.9%). Ever-treated patients had received teduglutide for a mean (SD) of 17.84 (20.01) months, at a mean (SD) daily dose of 0.05 (0.01) mg/kg (ranging between 0.002–0.06 mg/kg) and total daily dose of 3.05 (1.04) mg at enrollment (Table 6). Most of the ever-treated patients (*n* = 442; 89.8%) were receiving teduglutide 7 days a week at enrollment.

## 4. Discussion

The SBS Registry provides comprehensive information on the demographics and characteristics of a large population of patients with SBS-IF. Data from the registry have been used to carry out the first multicenter and multinational real-world analysis of such patients with a 10-year follow-up period. This registry is a unique database with independent monitoring and quality data. This paper summarizes the methodology used for the registry analyses and describes the baseline characteristics of the enrolled adult patients; subsequent papers will describe outcomes and pediatric patient data.

The baseline characteristics of patients included in this registry analysis are generally consistent with previous clinical trials [24,25,26,27] and real-world studies [46,47,49,50]. However, the most frequently reported cause of SBS-IF in this registry was Crohn’s disease, followed by intestinal ischemia; this differs from clinical trials and real-world studies that reported conditions including post-surgical/surgical complications, vascular disease, mesenteric ischemia, and intestinal obstruction as the most common causes [25,26,27,46,47,49,50,51]. However, a previous clinical trial [24] and a multinational observational study [50] reported Crohn’s disease as the main underlying causes of SBS-IF. The proportion of patients who reported Crohn’s disease as their primary cause of SBS-IF in this analysis may have been affected by the higher proportion of patients recruited in Europe and the USA than other countries—because Crohn’s disease is known to be more prevalent in these two regions compared with other countries included in this registry [50,52]. The increased availability of therapies, including biological treatments, [53] may have also contributed to more patients with Crohn’s disease having survived extensive small and large bowel surgery leading to an increased proportion of these patients enrolled in the registry. Of patients with a stoma, the most common type reported in this registry was ileostomy, followed by jejunostomy. Previous clinical trials reported similar proportions of patients with an ileostomy or jejunostomy [24,25,26], whereas real-world studies varied between ileostomies [47,49] and jejunostomies [51] being reported more frequently, or did not distinguish between the two types of stoma [46].

Patients who received teduglutide were prescribed the approved dose of 0.05 mg/kg/day at baseline [11,40], as per previous clinical trials [24,25,26,27] and other real-world studies [46,47], and in line with teduglutide prescribing information and the summary of product characteristics [11,40]. However, variations in the daily dose that patients received were recorded; patients may have adjusted their dose depending on their condition—for example, they would likely have been recommended a dose reduction to 0.025 mg/kg if their creatine clearance was less than 50 mL/min [54].

When comparing ever-treated and never-treated cohorts, patient demographics, characteristics and history at enrollment were broadly similar, apart from the length of the small intestine, the proportion of patients with an ileostomy and/or jejunostomy, and the duration and volume of PN/IV received. The length of remaining small intestine was less in the ever-treated than in the never-treated patients even though the mean proportions of remnant colon lengths were similar. The shorter length of remaining small intestine in the ever-treated patients than in the never-treated patients is similar to that observed in a previous real-world study [51]. Compared with never-treated patients, ever-treated patients were more likely to have an ileostomy, had received PN/IV for a longer duration and required lower volumes of PN/IV fluids at enrollment. Differences identified between ever-treated and never-treated patients may have been influenced by clinician referral bias and the management approach. Adjustments to PN/IV support prior to the provision of teduglutide could also have contributed to ever-treated patients having more optimal treatment than the never-treated patients [40]. It should be acknowledged that differences in patient baseline characteristics could influence response to teduglutide, including changes in PN/IV requirements over the duration of the study [55,56].

Patient medical histories were similar between ever-treated and never-treated patients, including the proportion of patients who had colorectal polyps and/or intestinal obstruction; however, a greater proportion of never-treated patients had a history of colorectal cancer and/or malignancy of any other type than ever-treated patients. Because the primary safety outcome is the occurrence of colorectal cancer, it is important to be aware of baseline variances that could affect the development of colorectal cancer over the course of the study. Differences may reflect patient selection, considering contraindications for teduglutide. In this registry, patients had a long-lasting recurrence-free interval between colorectal cancer, or other malignancies, and teduglutide initiation or beginning the registry. This could be related to careful selection of participating patients and health care professionals adhering to published recommendations that patients with a potential risk of malignancy, particularly those with a history of malignancies in the gastrointestinal tract, should not receive intestinal growth factors [2]. Differences in the baseline characteristics between ever-treated and never-treated patients suggest that patients with certain characteristics, such as a shorter length of remaining small intestine and longer duration of time receiving PN/IV, are more likely to be selected to receive teduglutide. Variations may also be indicative of differences in the monitoring of patients—for example, patients receiving teduglutide may receive more colonoscopies [11,40,41,42], leading to an increased number of polyps being identified, whether benign or potentially malignant. However, a similar proportion of ever- and never-treated patients were reported to have polyps in this registry.

Now that all patients have been recruited, interim analyses of safety and effectiveness outcomes will be performed throughout the minimum 10-year follow-up period. The registry will help to increase our understanding of the natural history and clinical management of SBS-IF in a real-world setting. The registry was open to all patients with SBS-IF who had been on PN/IV for at least 6 months and was not limited to centers that participated in teduglutide clinical trials, so as to address the potential limitations associated with studying a rare disease. As a multicenter, multinational study with broad inclusion criteria, this registry provides a large data set and long follow-up period for a rare disease and will produce more data on safety and effectiveness of teduglutide.

Limitations associated with this registry include the potential for recruitment or monitoring bias among sites because data were collected from multiple centers across several countries. Although a global representation was sought, over one-third of the data were collected from US centers and one-quarter from French centers. Additionally, no patients were recruited from Asia, so overall results may be biased toward certain populations. Because participation in the registry is voluntary, data may be biased toward individuals who choose to volunteer, who may differ from those who would not. Referral bias may have affected who was selected to receive teduglutide because the therapy was typically provided to patients with a shorter bowel, and therefore had more severe disease. As discussed above, this bias may be reflected in some baseline data; for instance, patients in the ever-treated group generally have a shorter remaining small intestine than those in the never-treated group. Having a 10-year follow-up period may result in patient drop off and losses to follow-up, which could result in selection bias in long-term follow-up data. As an observational study, unmeasured patient and clinical variables may confound the study results. By necessity, the measurement of remnant small intestine was based on a clinician’s estimate: intra-operative measurements and radiologic quantitation may differ and the state of contraction of the intestine could also alter the quantitation, both of which may result in a variability of measurements. Differences in monitoring practices between centers may also be present; for instance, the frequency of colonoscopies. Colonoscopy data will be reported in the final analysis of this study. Finally, International Classification of Diseases, 10th Revision-Clinical Modification codes were expanded in 2023 to include codes for SBS (K90.82) and IF (K90.83) [57,58]. Although the inclusion of these codes will create consistency in reporting, patients enrolled in this registry were identified before these codes were defined.

## 5. Conclusions

This is the first descriptive report of the baseline characteristics of adult patients enrolled in the SBS Registry and provides a global overview of a large cohort of patients with SBS-IF. Although patients who received (ever-treated) or who had never (never-treated) received teduglutide typically had similar baseline characteristics, differences identified between the two groups reflect potential variations in patients being selected to receive or not receive teduglutide as well as in the levels of monitoring of the two groups. Future data from this registry on the safety and effectiveness of teduglutide in a real-world clinical setting will benefit clinicians and patients by enabling better-informed treatment decisions in the management of SBS-IF.

## Figures and Tables

**Table 1:** Location of SBS Registry study sites and the number of patients who enrolled (per protocol set).

Registry Study Site Location	Number of Patients, *n* (%) (*N* = 1406)
Argentina	26 (1.8)
Australia	13 (0.9)
Canada	9 (0.6)
Croatia	2 (0.1)
Czech Republic	8 (0.6)
Denmark	49 (3.5)
Finland	1 (0.1)
France	340 (24.2)
Germany	191 (13.6)
Italy	69 (4.9)
Norway	16 (1.1)
Slovenia	8 (0.6)
Spain	26 (1.8)
Sweden	13 (0.9)
Switzerland	0 (0.0)
United Kingdom	83 (5.9)
United States of America	552 (39.3)

SBS, short bowel syndrome.

**Table 2:** Patient demographics and baseline characteristics (per protocol set).

	Teduglutide, Ever-Treated (*n* = 677)	Teduglutide, Never-Treated (*n* = 729)
**Age at enrollment**, years		
Mean (SD)	53.6 (15.49)	57.1 (15.26)
**Gender**, *n* (%)		
Women	403 (59.5)	443 (60.8)
**Race**, *n* (%)	*n* = 654	*n* = 696
White	498 (76.1)	515 (74.0)
**Ethnicity**, *n* (%)		
Hispanic	63 (9.3)	66 (9.1)
Non-Hispanic	439 (64.8)	443 (60.8)
Not available	175 (25.8)	220 (30.2)
**Height**, cm	*n* = 619	*n* = 685
Mean (SD)	166.7 (10.12)	167.0 (10.11)
**Weight**, kg	*n* = 637	*n* = 698
Mean (SD)	62.8 (15.09)	64.8 (15.32)
**BMI**, kg/m^2^	*n* = 609	*n* = 678
Mean (SD)	22.5 (4.68)	23.1 (4.73)
**Highest level of education**, *n* (%)	*n* = 675	*n* = 728
No formal education	7 (1.0)	7 (1.0)
Primary school not finished ^a^	4 (0.6)	1 (0.1)
Completed primary school ^a^	33 (4.9)	47 (6.4)
Secondary school not finished ^b^	28 (4.1)	36 (4.9)
Completed secondary school ^b^	160 (23.6)	191 (26.2)
Partial university education ^c^	59 (8.7)	51 (7.0)
Two-year university education	23 (3.4)	31 (4.3)
Four-year university education ^d^	73 (10.8)	62 (8.5)
Master’s degree	22 (3.2)	22 (3.0)
PhD/doctorate	10 (1.5)	7 (1.0)
Other	151 (22.3)	209 (28.7)
Unknown/not reported	105 (15.5)	64 (8.8)
**Employment status**, *n* (%)	*n* = 675	*n* = 729
Employed	117 (17.3)	88 (12.1)
Self-employed	20 (3.0)	26 (3.6)
Out of work for <1 year	7 (1.0)	7 (1.0)
Out of work for ≥1 year	51 (7.5)	58 (8.0)
Homemaker	24 (3.5)	19 (2.6)
Student	14 (2.1)	9 (1.2)
Retired	160 (23.6)	241 (33.1)
Unable to work	140 (20.7)	160 (21.9)
Unknown/not reported	144 (21.3)	121 (16.6)

^a^ Grades K–8. ^b^ Grades 9–12. ^c^ 1–3 years. ^d^ Bachelor of Arts or Bachelor of Science. BMI, body mass index; PhD, Doctor of Philosophy; SD, standard deviation.

**Table 3:** Patient history at enrollment (per protocol set).

	Teduglutide, Ever-Treated (*n* = 677)	Teduglutide, Never-Treated (*n* = 729)
**Age at onset/diagnosis of SBS-IF ^a^**, years	*n* = 610	*n* = 708
Mean (SD)	44.6 (18.55)	49.5 (17.95)
**Time between onset/diagnosis of SBS-IF and enrollment**, years	*n* = 619	*n* = 709
Mean (SD)	8.88 (9.94)	7.58 (9.12)
**Duration between last surgical resection and enrollment**, years	*n* = 629	*n* = 716
Mean (SD)	6.9 (7.69)	6.4 (7.93)
**Cause of major intestinal resection ^b^**, *n* (%)	*n* = 675	*n* = 729
Crohn’s disease	230 (34.1)	149 (20.4)
Intestinal ischemia ^c^	104 (15.4)	115 (15.8)
Intestinal volvulus	53 (7.9)	37 (5.1)
Surgical complications	26 (3.9)	48 (6.6)
**Length of remaining small intestine**, cm	*n* = 542	*n* = 602
Mean (SD)	85.3 (55.54)	101.0 (73.17)
**Colon remnant status**, *n* (%)	*n* = 675	*n* = 729
Intact colon	139 (20.6)	167 (22.9)
Partial colectomy	343 (50.8)	405 (55.6)
Total colectomy	193 (28.6)	157 (21.5)
**Proportion of remnant colon**, %	*n* = 192	*n* = 215
Mean (SD)	49.2 (24.01)	56.7 (21.67)
**Presence of stoma ^d^**, *n* (%)	*n* = 671	*n* = 727
Any stoma	358 (53.4)	417 (57.4)
Ileostomy	219 (61.2)	193 (46.3)
Jejunostomy	98 (27.4)	164 (39.3)
Colostomy	57 (15.9)	81 (19.4)
Other	10 (2.8)	32 (7.7)
**Intestinal transplant**	*n* = 675	*n* = 729
*n* (%)	10 (1.5)	10 (1.4)

^a^ Calculation includes patients diagnosed before 1 year of age. These patients were included with an age of 0. ^b^ Reported by >5% of total patients. ^c^ Of known or unknown etiology. ^d^ A patient may have multiple stoma types. SBS-IF, short bowel syndrome and intestinal failure; SD, standard deviation.

**Table 4:** Patient medical history at enrollment (per protocol set).

	Teduglutide, Ever-Treated (*n* = 677)	Teduglutide, Never-Treated (*n* = 729)
**Colorectal cancer**	*n* = 664	*n* = 724
All cases, *n* (%)	12 (1.8)	45 (6.2)
Duration between colorectal cancer end and teduglutide initiation or study inclusion, months ^a,b^	*n* = 5	*n* = 23
Mean (SD)	94.2 (30.5)	62.2 (58.8)
Range	67.9–144.3	2.1–242.9
**Malignancy of any other type**		
Yes, *n* (%)	120 (17.7)	219 (30.0)
Unknown, *n* (%)	13 (1.9)	13 (1.8)
Duration between malignancy end and teduglutide initiation or study inclusion, months ^a,c^	*n* = 34	*n* = 78
Mean (SD)	56.8 (62.3)	82.4 (93.6)
Range	0.3–277.2	0.0–505.1
**Colorectal polyps**, *n* (%)		
Yes	18 (2.7)	22 (3.0)
Adenoma	7 (1.0)	14 (1.9)
Non-adenoma	7 (1.0)	2 (0.3)
Unknown	4 (0.6)	6 (0.8)
**Other benign neoplasia**, *n* (%)		
GI tract ^d^	15 (2.2)	22 (3.0)
Hepatobiliary system	8 (1.2)	3 (0.4)
Pancreas	2 (0.3)	6 (0.8)
**Intestinal obstruction**, *n* (%)	199 (29.4)	242 (33.2)
**Pancreatic disease (other than neoplasia)**, *n* (%)	53 (7.8)	65 (8.9)
**Biliary disease (other than neoplasia)**, *n* (%)	75 (11.1)	94 (12.9)
**Motility disorder/pseudo-obstruction intestinal** **failure**, *n* (%)	44 (6.5)	73 (10.0)
**IF-associated liver disease**, *n* (%)	78 (11.5)	79 (10.8)
**Other GI surgery ^e^**, *n* (%)	365 (53.9)	445 (61.0)
**Central line infections in the last 12 months**, *n* (%)	135 (19.9)	164 (22.5)

A patient may have multiple medical conditions. For ever-treated and never-treated patients who participated in previous teduglutide clinical trials, baselines from the previous trials were used as the patient baselines for medical history data in this registry. For ever-treated patients who received teduglutide before registry enrollment but outside of a clinical trial or extension study, baseline for medical history data was the day of their first dose of teduglutide. ^a^ Patients could have more than one colorectal cancer/malignancy reported. Where multiple colorectal cancers/malignancies were recorded, the last record was used. The end dates were recorded based on investigator perception of when patients no longer had colorectal cancer/a malignancy of any type. ^b^ In cases where colorectal cancer or malignancy end dates were not available, start dates were used (*n* = 8 patients). ^c^ In cases where malignancy end dates were not available, start dates were used (*n* = 41 patients). Cases of colorectal cancers were excluded. ^d^ Other than colorectal polyps. ^e^ GI surgery received unrelated to SBS-IF. GI, gastrointestinal; IF, intestinal failure; SBS-IF, short bowel syndrome and intestinal failure; SD, standard deviation.

**Table 5:** Treatment received at baseline (effectiveness analysis set).

	Teduglutide, Ever-Treated ^a^ (*n* = 427)	Teduglutide, Never-Treated (*n* = 729)
**Volume PN/IV**, L/week, mean (SD)	*n* = 290 10.1 (6.64)	*n* = 634 12.4 (8.02)
**Frequency of received PN/IV**, days/week, mean (SD)	*n* = 291 5.3 (1.76)	*n* = 634 5.7 (1.78)

^a^ If teduglutide was first received post-enrollment, PN/IV use was recorded from the date immediately before the first dose of teduglutide and if patients were already receiving teduglutide when they entered the study, the PN/IV use reported in the 3 months before starting teduglutide was recorded. PN/IV, parenteral nutrition and/or intravenous fluids; SD, standard deviation.

**Table 6:** Patient teduglutide exposure at enrollment (per protocol set).

	Teduglutide, Ever-Treated (*n* = 677)
**Teduglutide exposure status**, *n* (%)	
Previously exposed	67 (9.9)
Currently exposed	442 (65.3)
Not available	168 (24.8)
**Teduglutide exposure**, *n* (%)	
Only during a clinical trial	10 (1.5)
During a clinical trial and outside of a clinical trial	26 (3.8)
Only outside of a clinical trial	473 (69.9)
Not available	168 (24.8)
**Duration of teduglutide use prior to enrollment**, weeks/months	*n* = 459
Mean (SD)	17.84 (20.01)
**Teduglutide daily dose**, mg/kg/day	*n* = 477
Mean (SD)	0.05 (0.01)
Range	0.002–0.06
**Total daily dose**, mg/day	*n* = 485
Mean (SD)	3.05 (1.04)
**Frequency of administration ^a^**, days/week, *n* (%)	*n* = 492
1	3 (0.6)
2	6 (1.2)
3	17 (3.5)
4	20 (4.1)
5	2 (0.4)
6	0 (0.0)
7	442 (89.8)

^a^ If more than one dose or frequency of teduglutide is reported, the most recent dose and frequency at enrollment was used. SD, standard deviation.

## Data Availability

The data sets, including the redacted study protocol, redacted statistical analysis plan, and individual participants’ data supporting the results reported in this article, will be made available from the completed study within 3 months from initial request to researchers who provide a methodologically sound proposal. The data will be provided after being de-identified, in compliance with applicable privacy laws, data protection, and requirements for consent and anonymization.

## Supplementary Materials

The following supporting information can be downloaded at: https://www.mdpi.com/article/10.3390/nu16152513/s1, Table S1: Inclusion and exclusion criteria for the SBS Registry; Supplementary Materials S1–S3 (S1: summary of adverse event reporting; S2 and S3: assumptions made for the simulation study, and calculations for the rates of annual attrition and percentages of new teduglutide patients who would enroll in the study).
