# Supplementary material for: Baseline Characteristics of Adult Patients Treated and Never Treated with Teduglutide in a Multinational Short Bowel Syndrome and Intestinal Failure Registry
Source: Nutrients. 2024 Aug 1;16(15):2513. doi: 10.3390/nu16152513 (PMC11314329; doi:10.3390/nu16152513)
Supplement: Supplementary file 1 [file nutrients-16-02513-s001.zip › nutrients-3050577-supplementary.pdf]

Supplementary Tables

Supplementary Table S1. Inclusion and exclusion criteria for the SBS Registry.

Inclusion	Exclusion
• Male or female<br>• Any age<br>• Diagnosis of SBS-IF<br>• Signed informed consent by the patient or a legally acceptable representative<br>• Medical record release by the patient or a legally acceptable representative<br>• Patients who have never received teduglutide must be on PN/IV support for ≥ 6 months at the time of enrollment	• Current participation in blinded clinical trial or their extension study<br>• Patients who have never received PN/IV<br>• Current or previous exposure to any GLP-2 analogs other than teduglutide

GLP-2, glucagon-like peptide 2; PN/IV, parenteral nutrition and/or intravenous fluids; SBS-IF, short bowel syndrome and intestinal failure.

Supplementary Materials S1

Adverse events (AEs) included:

- worsening (change in nature, severity, or frequency) of conditions present when the patient entered the study
- intercurrent illnesses
- drug interactions
- events related to or possibly related to concomitant medications
- prospective abnormal laboratory values (this includes significant shifts from baseline, within the range of normal, that the investigator considers to be clinically important)
- clinically significant abnormalities in physical examination, vital signs, weight, and medical tests and procedures
- lack of therapeutic effect.

In general, all AEs are collected from the time the informed consent is signed until the end of the observational period.

An investigator made the assessment of severity for each AE. The severity of AEs was recorded during the event, including the start and stop dates for each change in severity. An event that changed in severity was captured as a new event. Worsening of pretreatment events, after initiation of teduglutide, was recorded as a new AE. The medical assessment of severity was determined by using the following definitions.

- Mild: a type of AE that is usually transient and may require only minimal treatment or therapeutic intervention. The event does not generally interfere with usual activities of daily living.
- Moderate: a type of AE that is usually alleviated with specific therapeutic intervention. The event interferes with usual activities of daily living, causing discomfort, but poses no significant or permanent risk of harm to the patient.
- Severe: a type of AE that interrupts usual activities of daily living, or significantly affects clinical status, or may require intensive therapeutic intervention.

A physician/investigator made the assessment of relationship to teduglutide treatment (i.e. “treatment-related” or not) for each AE. Causality relationships were as follows.

- Related: the temporal relationship between the event and the administration of the medicinal product is compelling and/or follows a known or suspected response pattern

to that product, and the event cannot be explained by the patient’s medical condition, other therapies, or accident.

- Not related: the event can be readily explained by other factors such as the patient’s underlying medical condition, concomitant therapy, or accident, and there is no plausible temporal or biologic relationship between the medicinal product and the event.

An AE of special interest is an AE (serious or nonserious) of scientific and medical concern specific to the sponsor’s product or program and for which ongoing monitoring and immediate sponsor notification is required. The AEs of special interest that required expedited regulatory reporting included the following.

- Growth of pre-existing polyps of the colon.
- Benign neoplasia of the gastrointestinal tract, including the hepatobiliary system.
- Tumor-promoting ability (e.g. benign and/or malignant neoplasia of any kind, not limited to that of the gastrointestinal or hepatobiliary system).

A serious adverse event (SAE) is any untoward medical occurrence (whether considered to be related to medicinal product or not) that at any dose:

- results in death.
- is life-threatening.
    - note: the term “life-threatening” in the definition of “serious” refers to an event in which the patient was at risk of death at the time of the event; it does not refer to an event that hypothetically might have caused death had it been more severe.
- requires inpatient hospitalization or prolongation of existing hospitalization.
    - note: hospitalizations that are the result of elective or previously scheduled surgery for pre-existing conditions, and that have not worsened after initiation of treatment, should not be classified as SAEs. For example, admission for a previously scheduled ventral hernia repair would not be classified as an SAE; however, complication(s) resulting from hospitalization for an elective or previously scheduled surgery that meet(s) serious criteria must be reported as SAE(s).
- results in persistent or significant disability/incapacity.
- is a congenital abnormality/birth defect.
- is an important medical event.

Supplementary Materials S2

For the simulation study, the following assumptions were made.

- Five years of enrollment and a minimum of 10 years of follow-up for each patient.
- Annual attrition follows an exponential distribution.
- Enrollment follows a uniform distribution within each year of the 5-year enrollments.
- Approximately 60% of patients treated with teduglutide will have some remnant colon and be at risk for colorectal cancer.
- Approximately one-third of patients with short bowel syndrome and intestinal failure (SBS-IF) will have inflammatory bowel disease and experience double the risk of colorectal cancer as the general population. Overall, this means that the newly treated teduglutide population will have approximately one-third greater risk than the general population.
- The age distribution of patients entering the study will be similar to the age distribution of teduglutide clinical studies. The age distribution of patients with SBS-IF was 31.4%, 53.5%, and 15.1% for ages < 45, 45 to 65, and > 65 years, respectively. To simulate this age distribution, a scaled beta distribution was used with parameters a = 3.3 and b = 3.3.

Supplementary Materials S3

Based on the assumptions in **Supplementary Materials S1**, the rates of annual attrition (5%, 10%, 15%, 25%, and 50%) and the percentages of new teduglutide patients who would enroll in the study (20%, 30%, 40%, 50%, 60%, 70%, 80%, and 90%) were varied. For each scenario, the following was calculated.

- The expected number of person-years (PYs) accrued.
- The expected background incidence of colorectal cancer, based on Surveillance, Epidemiology and End Results (SEER) 2005–2009 colorectal cancer rates.
- The expected number of patients with colorectal cancer, based on the background SEER incidence and PYs accrued.
- The relative risk that could be ruled out with 80% power, which is the minimum number of colorectal cancer cases that would be required to conclude increased risk.
